# The Arabidopsis spliceosomal protein SmEb modulates ABA responses by maintaining proper alternative splicing of *HAB1*

**DOI:** 10.1007/s44154-021-00006-1

**Published:** 2021-08-18

**Authors:** Yechun Hong, Juanjuan Yao, Huazhong Shi, Yunjuan Chen, Jian-Kang Zhu, Zhen Wang

**Affiliations:** 1 Shanghai Center for Plant Stress Biology and Center for Excellence in Molecular Plant Sciences, Chinese Academy of Sciences, 200032 Shanghai, China; 2 University of Chinese Academy of Sciences (CAS), Shanghai, 200032 China; 3 Department of Chemistry and Biochemistry, Texas Tech University, Lubbock, TX 79409 USA

**Keywords:** ABA signaling, Alternative splicing, Cotyledon greening, HAB1, SmEb

## Abstract

Abscisic acid (ABA) signaling is critical for seed germination and abiotic stress responses in terrestrial plants. Pre-mRNA splicing is known to regulate ABA signaling. However, the involvement of canonical spliceosomal components in regulating ABA signaling is poorly understood. Here, we show that the spliceosome component Sm core protein SmEb plays an important role in ABA signaling. *SmEb* expression is up-regulated by ABA treatment, and analysis of Arabidopsis *smeb* mutant plants suggest that SmEb modulates the alternative splicing of the ABA signaling component HAB1 by enhancing the *HAB1.1* splicing variant while repressing *HAB1.2*. Overexpression of *HAB1.1* but not *HAB1.2* rescues the ABA-hypersensitive phenotype of *smeb* mutants. Mutations in the transcription factor ABI3, 4, or 5 also reduce the ABA hypersensitivity of *smeb* mutants during seed germination. Our results show that the spliceosomal component SmEb plays an important role in ABA regulation of seed germination and early seedling development.

Alternative splicing occurs in many genes involved in diverse processes during plant development and responses to abiotic stresses (Staiger and Brown 2013). mRNA splicing is mainly mediated by the spliceosome consisting of five conserved small nuclear ribonucleoproteins (snRNPs) in eukaryotes. The seven Sm core proteins (B/B′, D1, D2, D3, E, F and G) are common components of the five snRNPs and are essential for their assembly (Reddy 2007; Wahl et al. 2009). Among the Sm core proteins, SmEb has been reported to positively regulate cold stress response by modulating pre-mRNA splicing (Capovilla et al. 2018). In this study, we found that the transcript level of *SmEb* was induced by ABA treatment (Fig. 1a), which suggests that SmEb might be involved in ABA response. To uncover the molecular mechanism underlying SmEb-modulated ABA response, we first characterized two T-DNA insertion mutant alleles of *SmEb*, designated *smeb-1* and *smeb-2*. The *smeb-1* and *smeb-2* mutants have T-DNA insertions in the second exon and fourth intron of the At2g18740 gene, respectively (Fig. 1b). Quantitative reverse transcription-PCR (qRT-PCR) analysis determined that the transcript level of *SmEb* was nearly undetectable in *smeb-1* and *smeb-2* mutants (Fig. 1c). Phenotyping of *smeb-1* and *smeb-2* showed that these two mutants were much more sensitive to ABA treatment than the Col-0 wild type plants in cotyledon greening during seed germination (Fig. 1d). Under normal growth conditions, the rates of cotyledon greening were not different between *smeb* mutants and wild type plants (Fig. 1e). However, with 0.5 μM ABA supplemented in the medium, the emerged seedlings with green cotyledons in *smeb-1* and *smeb-2* mutants were only approximately 67% and 54%, in contrast to the near 100% green cotyledons in the wild type at the seventh day of the treatment (Fig. 1f). In addition, compared with the ~ 52% green cotyledon rate in the wild type, only approximately 8.3% of *smeb-1* and 6.5% of *smeb-2* mutant cotyledons turned green after 1 μM ABA treatment for 7 days (Fig. 1g). Together, these results indicate that SmEb is a positive regulator of ABA responses in Arabidopsis.

**Fig. 1:**
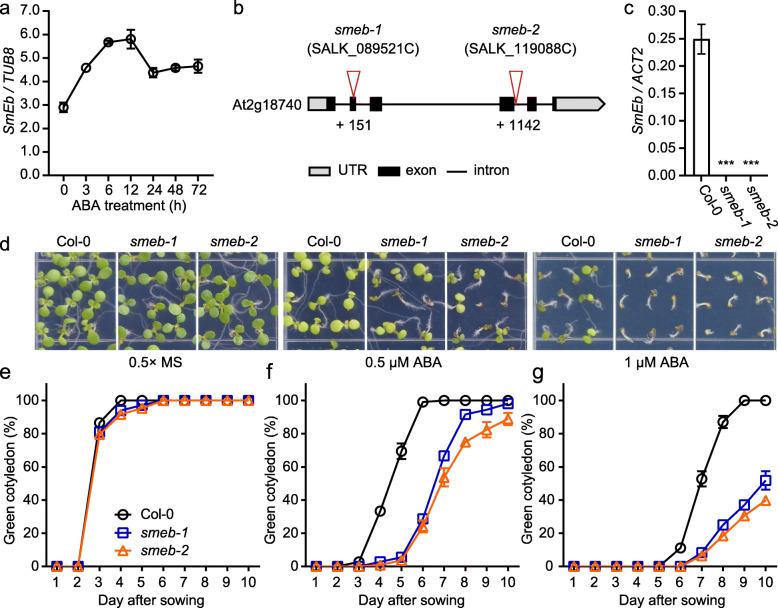
*SmEb* is involved in ABA responses during seed germination and cotyledon greening. **a** The transcription levels of *SmEb* in 7-day-old wild-type (Col-0) seedlings after exogenous ABA treatment for 0, 3, 6, 12, 24, 48, or 72 h. The transcript levels of *TUB 8* were used as an internal control. **b** Schematic diagram showing the T-DNA insertions in the *smeb* mutants. **c** Quantitative RT-PCR analysis of *SmEb* transcript levels in Col-0, *smeb-1*, and *smeb-2* mutant plants. **d** 7-day-old seedlings of Col-0, *smeb-1*, and *smeb-2* grown in 0.5× MS medium containing 0, 0.5, and 1 μM ABA. **e-g** Percentages of green cotyledon of Col-0, *smeb-1*, and *smeb-2* after seed sowing for indicated days in 0.5× MS medium with different concentrations of ABA. Error bars represent the ± SD (*n* = 3 biological repeats). ***P < 0.001, Student’s t-test

The core ABA signaling pathway is initiated by ABA binding to the PYR/PYL/RCAR receptors, which then interact with and inhibit the type 2C protein phosphatases (PP2Cs) co-receptors such as HYPERSENSITIVE TO ABA1 (HAB1) (Ren et al. 2017; Gong et al. 2020). HAB1 serves as a negative regulator for ABA signaling in seed germination and cotyledon greening, and previous studies have shown that the transcriptional up-regulation of *HAB1* is critical for ABA signaling (Melcher et al. 2009; Wang et al. 2018a). As a result, suppression of the accumulation of *HAB1* transcripts leads to the activation of ABA signal transduction in plants. Recent reports have shown that splicing factors play critical roles in plant response to ABA through regulating the splicing of *HAB1* transcripts (Wang et al. 2015; Zhan et al. 2015; Bi et al. 2021). However, it is unclear whether the canonical spliceosomal component SmEb may contribute to the regulation of *HAB1* pre-mRNA splicing and ABA responses. To determine the role of SmEb in the regulation of ABA signaling, we first examined the transcripts of *HAB1* by using RT-PCR and Sanger sequencing. As shown in Fig. 2a, alternatively spliced forms of *HAB1* transcripts, *HAB1.1* to *1.5*, were identified under normal and ABA treatment conditions. Only two types of HAB1 polypeptides were encoded by these five spliced forms. The *HAB1.1*, *HAB1.3*, and *HAB1.4* transcripts are translated into full-length HAB1 protein, named as HAB1.1, while the *HAB1.2* and *HAB1.5* transcripts produce a truncated HAB1, designated as HAB1.2 (Fig. 2b). Under normal growth conditions, the transcript levels of the five *HAB1* spliced forms showed no difference between *smeb* mutants and Col-0 wild type plants (Fig. 2c - g). However, the transcript levels of all *HAB1* variants in *smeb* mutants were significantly reduced after ABA treatment for 6 h when compared with those of the Col-0 wild type. After 12 h of ABA treatment, the accumulation of *HAB1.1*, *1.3*, *1.4*, and *1.5* transcripts were clearly lower in *smeb* mutants than in Col-0 wild type, whereas the transcript level of *HAB1.2* was higher in *smeb* mutants than in Col-0 wild type plants (Fig. 2c - g). Since the expression level of *HAB1.3* is very low compared with the other four variants (Fig. 2e), and the HAB1.1 and HAB1.2 proteins function antagonistically (Wang et al. 2015), it is likely that the reduction of *HAB1.1* and *HAB1.4* transcripts and thus the decreased level of HAB1.1 protein contributes to the ABA hypersensitive phenotype of *smeb* mutants.

**Fig. 2:**
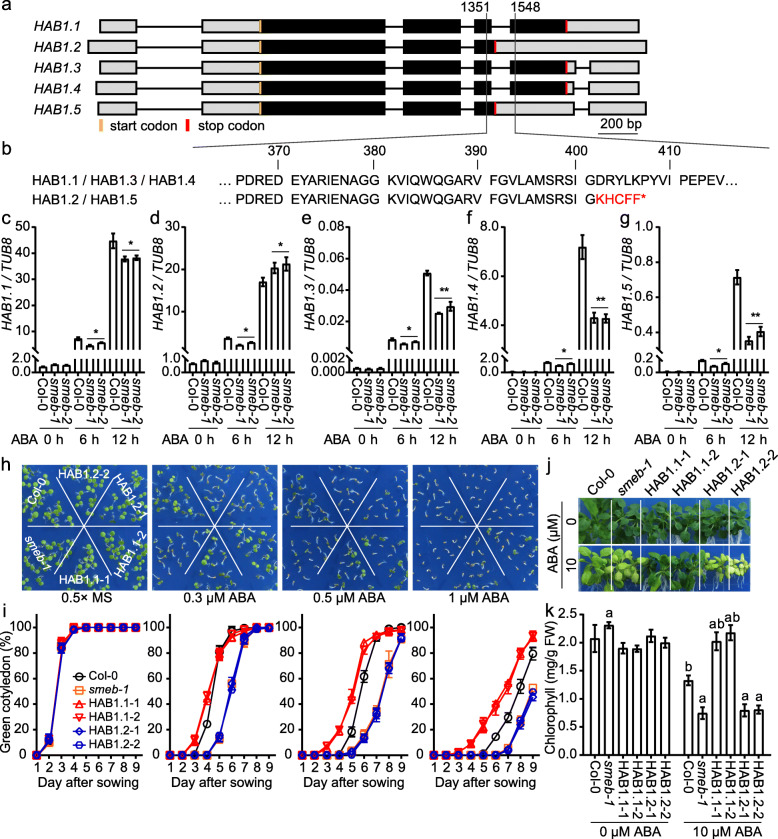
SmEb modulates the alternative splicing of *HAB1* in response to ABA. **a** Different alternative splicing variants of *HAB1* as determined by sequence analysis of cloned PCR products. The lines between boxes indicate introns. The grey and black boxes represent the UTR and exon, respectively. **b** Amino acid sequence resulting from alternative splicing and the formation of a full-length and truncated HAB1 proteins in the close-up view. *HAB1.1*, *HAB1.3* and *HAB1.5* encode the full-length HAB1 protein, while the *HAB1.2* and *HAB1.4* encode the truncated HAB1 protein. **c-g** Relative transcript levels of different alternative splicing variants of *HAB1* in Col-0, *smeb-1* and *smeb-2* plants subjected to 50 μM ABA for 0, 6, or 12 h, as determined by qRT-PCR analysis. Values are mean ± SD (*n* = 3), * *P* < 0.05, ** *P* < 0.01, Student’s t-test. **h** Cotyledon greening assay of Col-0, *smeb-1*, HAB1.1-1, HAB1.1-2, HAB1.2-1, and HAB1.2-2 after seed sowing for 6 days in 0.5× MS medium containing 0, 0.3, 0.5, or 1 μM ABA. HAB1.1-1 and HAB1.1-2, two independent lines of *smeb-1* expressing 3*5S:HAB1.1-3 × FLAG* transgene. HAB1.2-1 and HAB1.2-2, two independent lines of *smeb-1* expressing 3*5S:HAB1.2-3 × FLAG* transgene. **i** Percentages of green cotyledon of Col-0, *smeb-1*, HAB1.1-1, HAB1.1-2, HAB1.2-1, and HAB1.2-2 after seed sowing for indicated days in 0.5× MS medium supplementing 0, 0.3, 0.5, or 1 μM ABA. Error bars represent ± SD (*n* = 6). **j** Seven-day-old Col-0, *smeb-1*, HAB1.1-1, HAB1.1-2, HAB1.2-1, and HAB1.2-2 seedlings grown in 0.5× MS medium were transferred to 0.5× MS medium with or without 10 μM ABA and grown for an additional 3 weeks. **k** The chlorophyll content of rosette leaves of Col-0, *smeb-1*, HAB1.1-1, HAB1.1-2, HAB1.2-1, and HAB1.2-2 plants shown in (**j**). Values are mean ± SD (*n* = 6). The letters a and b above the columns indicate a significant difference relative to Col-0 and *smeb-1* mutant, respectively (*P* < 0.05, Student’s t-test)

To test the hypothesis that the reduction of HAB1.1 protein is responsible for the ABA hypersensitive response phenotype of *smeb* mutants, we expressed the full-length cDNAs of *HAB1.1* and *HAB1.2* driven by the CaMV 35S promoter in *smeb-1* mutant plants. Two independent homozygous lines of *35S:HAB1.1-3 × FLAG/smeb-1* (designated as HAB1.1-1 and HAB1.1-2) and *35S:HAB1.2-3 × FLAG/smeb-1* (named as HAB1.2-1 and HAB1.2-2) were identified, and qRT-PCR analysis showed that the expression levels of *HAB1.1* and *HAB1.2* in the overexpression lines are approximately six- to nine-fold higher than the wild type controls. Cotyledon greening assays showed that overexpression of *HAB1.1* but not *HAB1.2* could fully rescue the hypersensitive phenotype of *semb-1* in response to ABA treatment (Fig. 2h and i). These results further support that *HAB1* is a downstream target of SmEb and decreased accumulation of HAB1.1 protein is responsible for the *smeb* mutant phenotype. The transgenic seedlings were further tested on media supplemented with 10 μM ABA. The results showed that the ectopic expression of *HAB1.1* but not *HAB1.2* also suppressed the chlorosis phenotype of *smeb-1* in response to ABA treatment (Fig. 2j and k). These results indicate that the *HAB1.1* but not the *HAB1.2* spliced form is the functional variant in SmEb-modulated ABA response, and it is very likely that the reduction of HAB1.1 protein contributes to the ABA-hypersensitive phenotype of *smeb-1* mutant plants.

Inhibition of the full length HAB1 protein results in the activation of SnRK2 protein kinases, leading to the activation of downstream transcription factors such as ABA INSENSITIVE (ABI) 3, ABI4, and ABI5 in the ABA signaling pathway (Feng et al. 2014; Zhu 2016). Although ABI3, ABI4, and ABI5 belong to different transcription factor families, they are all positive regulators in plant ABA responses (Skubacz et al. 2016). The genetic interaction between *SmEb* and *ABI3*, *ABI4*, and *ABI5* in modulating seed germination and cotyledon greening in response to ABA treatment was studied by generating *smeb-1* *abi3-8*, *smeb-1* *abi4-1*, and *smeb-1* *abi5-7* double mutant lines. Cotyledon greening assays revealed that the *abi3*, *abi4*, or *abi5* mutation could rescue the ABA-hypersensitive phenotype of *smeb-1* mutant plants (Fig. 3). However, among the three *abi* mutations, *abi3* showed weaker suppression of the *smeb-1* mutant phenotype (Fig. 3), suggesting that the effect of *smeb* mutation on ABA response is partially independent of *ABI3*. Taken together, it is likely that the *smeb-1* mutation causes reduced production of HAB1.1 and/or other PP2Cs protein, which leads to enhanced ABA signaling and activation of ABA responses that are dependent on the downstream transcription factors ABI3, 4, and 5, resulting in ABA-hypersensitivity of the *smeb-1* mutant.

**Fig. 3:**
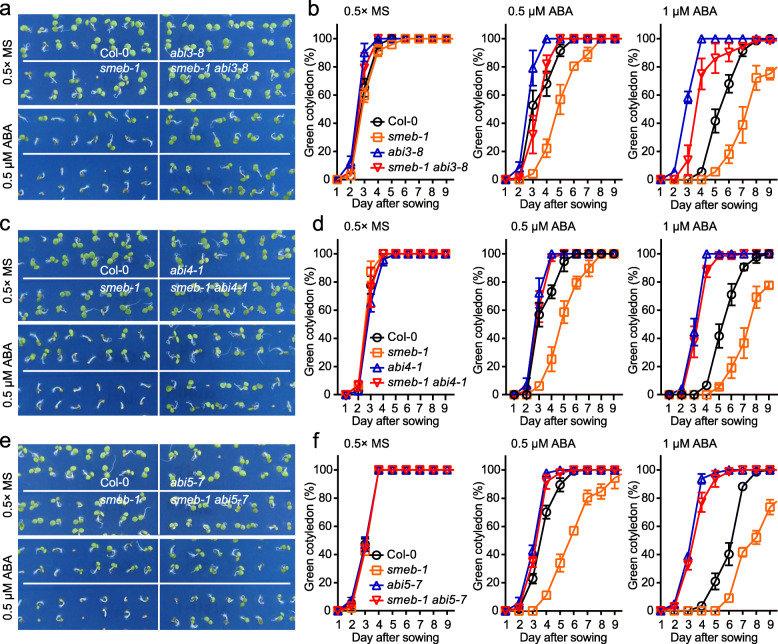
The *abi3*, *abi4* and *abi5* mutations suppress the ABA-hypersensitivity of *smeb* mutant. **a** Cotyledon greening assay of Col-0, *smeb-1*, *abi3-8*, and *smeb-1* *abi3-8* after seed sowing for 5 days in 0.5× MS medium with or without 0.5 μM ABA. **b** The percentage of green cotyledon of Col-0, *smeb-1*, *abi3-8*, and *smeb-1* *abi3-8* in response to ABA. Green cotyledons were recorded after seed sowing on medium supplemented with 0, 0.5, or 1 μM ABA. Error bars represent ± SD (*n* = 6). **c** Cotyledon greening assay of Col-0, *smeb-1*, *abi4-1*, and *smeb-1* *abi4-1* after seed sowing for 5 days in 0.5× MS medium with or without 0.5 μM ABA. **d** The percentage of green cotyledon of Col-0, *smeb-1*, *abi4-1*, and *smeb-1* *abi4-1* in response to ABA. Green cotyledons were recorded after seed sowing on medium containing 0, 0.5, or 1 μM ABA. Error bars represent ± SD (*n* = 6). **e** Cotyledon greening of Col-0, *smeb-1*, *abi5-7*, and *smeb-1* *abi5-7* after seed sowing for 5 days in 0.5× MS medium with or without 0.5 μM ABA. **f** The percentage of green cotyledon of Col-0, *smeb-1*, *abi5-7*, and *smeb-1* *abi5-7* after seed sowing for indicated days in 0.5× MS medium supplementing 0, 0.5, or 1 μM ABA. Error bars represent ± SD (*n* = 6)

In addition to ABA signaling, ABA biosynthesis plays a role in controlling plant ABA responses (Nakashima and Yamaguchi-Shinozaki 2013; Wang et al. 2018a). The de novo ABA biosynthesis pathway is well known in Arabidopsis, and the xanthoxin dehydrogenase encoded by *ABA DEFICIENT 2* (*ABA2*) is a critical enzyme for ABA biosynthesis (Seo and Koshiba 2002). To determine whether the ABA-hypersensitive phenotype of *smeb* mutants may be due to enhanced ABA accumulation, we measured ABA contents, and found that the two *smeb* mutants and Col-0 wild type had comparable cellular ABA levels in seedlings and dry seeds (Fig. 4a - c). This result indicates that the *smeb* mutations do not affect ABA biosynthesis. We further tested the genetic interaction between *aba2* and *smeb-1* by analyzing the double mutant *smeb-1* *aba2-1*. The *aba2* mutant is hyposensitive to ABA in seed germination (Lin et al. 2007). The results indicated that the *aba2* mutation could not rescue the ABA-hypersensitive phenotype of *smeb-1* mutant in cotyledon greening during seed germination (Fig. 4d - g). These results further support that SmEb modulates ABA responses by regulating ABA signaling rather than ABA accumulation.

**Fig. 4:**
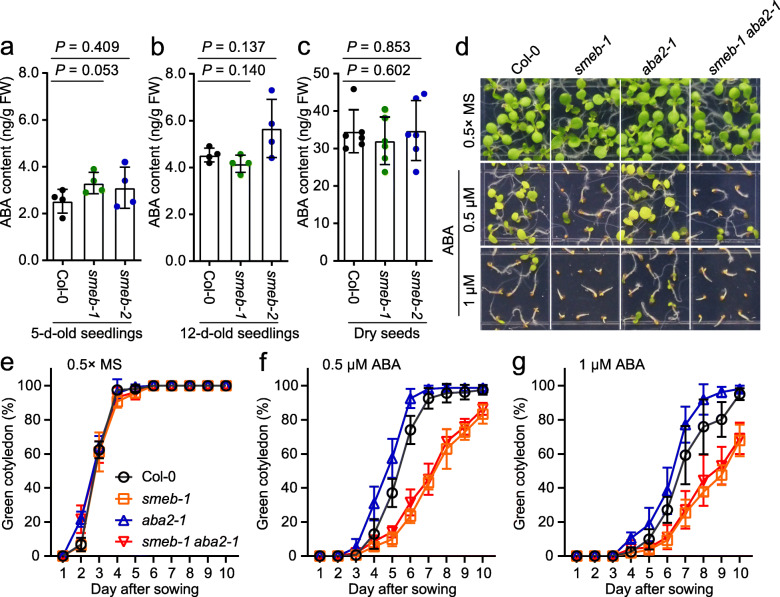
Effect of ABA biosynthesis on ABA sensitivity of *smeb-1* mutant. **a**, **b** The ABA contents were measured in seedlings of Col-0, *smeb-1*, and *smeb-2* seedlings under normal conditions after seed sowing for 5 (**a**) and 12 (**b**) days, respectively. Values are mean ± SD (*n* = 4). **c** The ABA contents in dry seeds of Col-0, *smeb-1*, and *smeb-2*. Error bars represent ± SD (*n* = 6), *P* values are determined by Student’s t-test. **d** Cotyledon greening assay of Col-0, *smeb-1*, *aba2-1* and *smeb-1* *aba2-1* after seed sowing for 7 days in 0.5× MS medium containing 0, 0.5, or 1 μM ABA. **e**-**g** The percentage of green cotyledon of Col-0, *smeb-1*, *aba2-1* and *smeb-1* *aba2-1* after seed sowing for indicated days in 0.5× MS medium supplementing 0, 0.5, or 1 μM ABA. Error bars represent ± SD (*n* = 6)

In conclusion, we have identified that SmEb, a core spliceosome component involved in pre-mRNA splicing, modulates the alternative splicing of *HAB1* that encodes an ABA co-receptor critical for ABA responses in Arabidopsis. Our results suggest that this regulatory mechanism helps to fine-tune ABA signaling during seed maturation, germination and cotyledon greening by maintaining proper levels of *HAB1* splicing variants in response to changes in endogenous ABA contents. When ABA concentration is low, SmEb is not critical for *HAB1* pre-mRNA splicing. When ABA concentration increases, *SmEb* expression is up-regulated, and SmEb becomes critical for maintaining a proper ratio of the two splicing variants that encode antagonistic HAB1 proteins, HAB1.1 and HAB1.2. Our findings highlight the crucial roles of mRNA splicing control of key genes in plant hormonal signaling and stress responses. Future work will determine how increased *SmEb* may affect the splicing of *HAB1* pre-mRNAs in response to ABA treatment.

## Materials and methods

### Plant materials and growth conditions

All Arabidopsis (*Arabidopsis thaliana*) plants used in this study are in the Columbia-0 background. The T-DNA insertion mutants, including *smeb-1* (SALK_089521C) and *smeb-2* (SALK_119088C), were obtained from the Arabidopsis Biological Resource Center (ABRC; The Ohio State University, Columbus, OH, USA). The *abi3-8*, *abi4-1*, *abi5-7*, and *aba2-1* mutants were collected from previous studies. The double mutants, including *smeb-1* *abi3-8*, *smeb-1* *abi4-1*, *smeb-1* *abi5-7*, and *smeb-1* *aba2-1*, were generated by genetic crossing and subsequent PCR-based genotyping in the F2 population. Seeds were surface-sterilized and stratified at 4 °C for 2 days, and then sown in 0.5× Murashige and Skoog (MS) medium plates and grown at 22 °C under 16 h light / 8 h dark photoperiod.

To construct the *HAB1.1* and *HAB1.2* transgenic plants, the coding sequence (CDS) of two *HAB1* splicing isoforms, *HAB1.1* and *HAB1.2*, were amplified and cloned into pCambia1305 binary vector (Hong et al. 2020) to obtain the *35S:HAB1.1-3 × FLAG* and *35S:HAB1.2-3 × FLAG* constructs, respectively. These two constructs were then respectively introduced into *smeb-1* mutant plants by using *Agrobacterium*-mediated floral dip method (Clough and Bent 1998). The seeds of T3 homozygous transgenic plants were identified for further analysis.

### Physiological phenotype assays

For seed germination assay, Arabidopsis seeds were surface-sterilized and stratified at 4 °C for 2 days before sowing on 0.5× MS medium plates containing indicated concentrations of ABA. The plates were kept in a growth chamber at 22 °C under 16 h light / 8 h dark conditions. The percentages of green cotyledon were calculated with three to six replicates for indicated days after sowing. For analysis of ABA sensitivity at post-germination stage, seven-day-old seedlings of Col-0, *smeb-1* and transgenic plants grown in 0.5× MS plates were transferred to 0.5× MS medium with or without 10 μM ABA for additional 3 weeks, and the chlorophyll content were then determined in rosette leaves. The average content of total chlorophyll was calculated from six independent experiments (*n* = 3 plants).

### Quantification of *HAB1* splice variants

The transcripts of *HAB1* gene were amplified by RT-PCR from the cDNA of 7-day-old Col-0 and *smeb* mutant seedlings after 50 μM ABA treatment for 12 h. The PCR products with three bands were cloned into the vector using pMD™18-T Vector Cloning Kit (TaKaRa), followed by Sanger sequencing. The *HAB1* alternative splicing variants were distinguished in qRT-PCR by using primer pairs that only amplify one of the five variants, which were designed according to the last three exons and 3’UTR of At1g72770. The primer pairs used to determine the transcript levels of the five splicing variants of *HAB1* by qRT-PCR are as follows. 5′-TCTAGGTCCATCGGTGACAGATATCT-3′ as forward primer of *HAB1.1*, *1.3*, and *1.4*; 5′-GCACATCACACAAACGTGGCTTG-3′ as forward primer of *HAB1.2* and *1.5*; 5′-TACGAAAACTCGAAACTTACCCCCA-3′ as reverse primer of *HAB1.1*; 5′-ACCTGTCGAAATTAGATCCTTAACGATCT-3′ as reverse primer of *HAB1.2*; 5′-TTTTCTTTATTTTTTTTACCCCCAGT-3′ as reverse primer of *HAB1.3*; and 5′-TTCTTTATTTTTTCTCCCTTCCCCAGTA-3′ as reverse primer of *HAB1.4* and *1.5*.

### Determination of chlorophyll and ABA content

The chlorophyll and ABA contents were determined as described in previous study (Wang et al. [Bibr CR16][Bibr CR17]). Briefly, to quantify chlorophyll contents, the chlorophyll was extracted from 100 mg rosette leaves with 0.6 mL 80% (v/v) acetone buffer. The extract was then centrifuged for 5 min at 4 °C, and the supernatant was collected and the absorbance of chlorophyll at wavelengths 645 and 663 nm was measured using NanoDrop spectrophotometer 2000C (Thermo Scientific). To determinate ABA contents, endogenous ABA was extracted from 50 mg fresh leaf tissue of each sample using 0.5 mL homogeneous buffer (70% methanol, 0.1% formic acid). 2 ng ABA-d6 (Olchemim, Olomouc, Czech) was added to the extracts as an internal standard. The mixture was diluted 2 times with deionized water, and the ABA content of 50 μL dilution of each sample was determined by the UPLC-Triple TOF 5600+ system (Sciex, Concord, Canada).

### Quantification of gene expression

Total RNAs were extracted from rosette leaves of 7-day-old seedlings grown in 0.5× MS liquid medium with or without 50 μM ABA treatment using Quick RNA Isolation Kit (Huayueyang). Reverse transcription was carried out using iScript cDNA synthesis kit (*Bio-RAD*), followed by quantitative PCR on a CFX96™ Real-Time system (*BIO-RA*D) with AceQ qPCR SYBR Green Master Mix (Vazyme Biotech co., ltd). The data of expression profiles were analyzed and illustrated by temporal changes using the Prism 7 (GraphPad Software). *TUB8* was used as an internal control.

## Data Availability

All data generated or analyzed during this study are included in this published article.

## Abbreviations

ABA: Abscisic acid
T-DNA: Transfer DNA
qRT-PCR: Quantitative reverse transcription-PCR
PP2C: Type 2C protein phosphatases
HAB1: HYPERSENSITIVE TO ABA1
ABI3: ABA INSENSITIVE 3
ABI4: ABA INSENSITIVE 4
ABI5: ABA INSENSITIVE 5
ABA2: ABA DEFICIENT 2
MS: Murashige and Skoog
